# Orthogeriatric co-management of fragility fractures: a retrospective cohort study on one-year mortality and functional outcomes across fracture types

**DOI:** 10.1186/s12877-025-06913-6

**Published:** 2025-12-18

**Authors:** Carlos Pankratz, Annika Risch, Jacob Oxen, Raffael Cintean, Florian Gebhard, Konrad Schuetze, Alexander Boehringer

**Affiliations:** Department of Trauma-, Hand-, Plastic- and Reconstructive Surgery, University Ulm Medical Centre, Albert-Einstein-Allee 23, 89081 Ulm, Germany

**Keywords:** Orthogeriatric co‑management, Fragility fractures, Geriatric trauma, One‑year mortality, Functional outcomes, Discharge destination

## Abstract

**Background:**

Fragility fractures in older adults are associated with high morbidity and mortality, yet evidence beyond hip fractures remains limited. This study analysed one‑year mortality and functional outcomes across multiple fracture types managed under structured orthogeriatric co‑management in a certified Geriatric Trauma Centre.

**Methods:**

We retrospectively reviewed 486 patients aged ≥ 70 years (mean 83.8 ± 6.6; 70.2% female) with fragility fractures (proximal and periprosthetic femur, cervical and thoracolumbar spine, pelvic ring, proximal humerus) treated between 2019 and 2021. All patients were screened using the Identification of Seniors at Risk (ISAR) and received interdisciplinary orthogeriatric care. Mortality and residential status at one year were ascertained via the national citizen registry, ensuring complete follow-up. Primary endpoints were one‑year mortality and residential status. Logistic regression identified predictors of survival.

**Results:**

Overall, one‑year mortality was 32.3%, highest for periprosthetic femoral (42.3%) and lowest for pelvic ring fractures (18.4%). Pelvic ring fractures independently predicted improved survival (OR 3.46; *p* = 0.006), while Charlson Comorbidity Index > 6 predicted higher mortality (OR 0.44; *p* = 0.001). Only 52.3% of previously independent patients maintained independent living after one year. Discharge home strongly correlated with survival (mortality 12.9% vs. 39.6% after nursing home discharge; *p* = 0.003).

**Conclusions:**

Fragility fractures of all types carry high one‑year mortality and substantial functional decline. Discharge destination is a powerful prognostic marker and should inform early rehabilitation planning. Orthogeriatric strategies must extend beyond hip fractures and integrate frailty assessment to identify *orthogeriatric responders* likely to regain independence.

## Background

Fragility fractures in older adults, caused by low-energy trauma, represent an increasing healthcare burden in ageing societies [1, 2]. In Germany alone, incidence rates for hip, humerus, vertebral body, and pelvic ring fractures rose by 10–39% between 2009 and 2019 [3]. These injuries are closely linked to significant morbidity, elevated mortality, and loss of independence, often marking a critical transition toward long-term dependency for older patients [4]. Despite this impact, current clinical research has largely focused on hip fractures, where orthogeriatric co-management is now widely regarded as standard of care. Meta-analyses and systematic reviews have demonstrated that such interdisciplinary models can reduce one-year mortality and significantly improve functional outcomes in hip fracture populations [5–7].

In contrast, evidence on orthogeriatric co-management remains limited for other common fragility fracture types, including pelvic ring, vertebral, proximal humerus, and periprosthetic femoral fractures. These injuries are increasingly recognized as clinically relevant, with recent cohort data demonstrating substantial in-hospital and one-year mortality rates that approach or even exceed those observed in hip fractures [8]. A large retrospective matched-cohort study using data from a publicly funded healthcare system demonstrated that fragility fractures at any anatomical site are associated with a significantly increased risk of mortality in adults aged over 65 years [9]. Recent data show that patients with immobilizing fragility fractures – including hip, thoracolumbar spine, and pelvic injuries – had significantly higher in-hospital and one-year mortality compared to those with non-immobilizing fragility fractures, although two-year mortality did not differ significantly between groups [10].

Frailty among geriatric patients is a multidimensional syndrome characterised by reduced physiological reserve and increased vulnerability to stressors, predisposing older adults to adverse outcomes after injury or surgery. It represents a key determinant of prognosis in geriatric trauma, influencing both short- and long-term recovery. Screening for frailty at hospital admission enables early risk stratification and targeted co-management within orthogeriatric pathways [11]. A variety of assessment tools have been developed to screen for frailty in clinical settings, ranging from brief screening instruments to multidimensional geriatric assessments. The Identification of Seniors at Risk (ISAR) score, a brief and validated tool [12, 13], has been shown to correlate with frailty and adverse outcomes, making it a pragmatic marker for identifying vulnerable older patients in acute trauma care [14, 15].

Despite these developments, there remains a significant gap in the literature, as few studies have systematically compared long-term outcomes across multiple fragility fracture types [9] or have been conducted within structured orthogeriatric co-management models [10, 16]. Even fewer have included residential status as a functional endpoint alongside survival, despite its relevance for older adults seeking to maintain independence after a fracture. The available studies focusing on this aspect primarily address hip fractures [17, 18]. Furthermore, many studies are limited by incomplete follow-up, especially regarding post-discharge outcomes, which can introduce significant bias [10].

To address these gaps, we conducted a retrospective cohort study at a certified Geriatric Trauma Centre (DGU^®^) in Germany. Leveraging the national citizen registry to achieve complete one-year follow-up for mortality and residential status, our study aims to provide robust, fracture-specific outcome data within a uniform care setting. We also aimed to identify independent predictors of mortality, including fracture type, comorbidity burden, dementia, and discharge destination. In addition, we introduce the concept of orthogeriatric responders – patients who regain pre-fracture independence under structured orthogeriatric co-management – to highlight the importance of functional recovery as a complementary outcome alongside survival. Hip fractures were thereby intentionally included, as they represent the largest and best-established fragility fracture group and provide a consistent reference for comparison with other fracture types.

## Methods

**Fig. 1:**
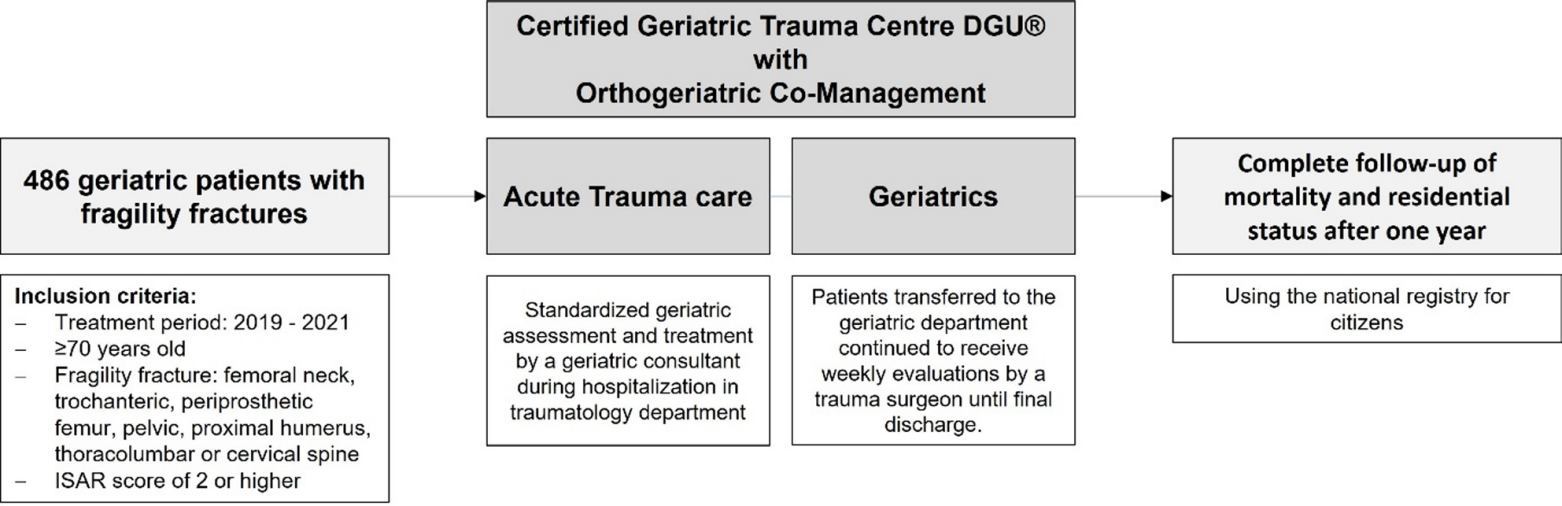
Study design flow chart. *DGU: Deutsche Gesellschaft für Unfallchirurgie (German Society for Trauma Surgery); ISAR: Identification of Seniors at Risk*

We retrospectively evaluated the data of 486 patients (83.8 ± 6.6 years; 145 men, 341 women) with fragility fractures – including proximal and periprosthetic femur, cervical and thoracolumbar spine, pelvic ring, and proximal humerus fractures – who received orthogeriatric co-management at our certified Geriatric Trauma Centre DGU^®^ during the years 2019 to 2021, following the centre’s initial certification in 2019. Inclusion criteria comprised patients aged over 70 years with an ISAR score of 2 or higher. Presented data were obtained with institutional and local ethical committee approval for the use of the data (Fig. 1 Study design flow chart Figure 1).

At our Level 1 trauma centre, all patients aged ≥ 70 years with a fragility fracture are routinely screened using the ISAR score on the first day of admission. Patients with an ISAR score of 2 or higher undergo standardized geriatric assessment and receive specialized geriatric treatment provided by a geriatric consultant during their hospital stay in the traumatology department. This comprehensive early geriatric rehabilitative care includes regular interdisciplinary meetings and the development of individualized rehabilitation goals with a focus on managing geriatric syndromes.

Patient discharge is coordinated by social workers, attending surgeons, and geriatric consultants. Patients discharged from the acute trauma centre to geriatrics are continuously monitored with weekly visits by an experienced trauma surgeon. The orthogeriatric care program includes occupational therapy, physiotherapy, nutritional counselling, sports therapy, and continuous supervision by both geriatricians and orthopaedic trauma surgeons.

The collected parameters included demographic data, fracture type, surgical and non-surgical complications, discharge modality, residential status, and one-year mortality. Surgical complications were defined as events requiring operative revision, such as hematoma, bleeding, surgical site infection, implant failure, or fracture displacement. Non-surgical complications included major cardiovascular events (e.g., thrombosis, embolism, myocardial infarction, stroke), infectious complications (e.g., pneumonia, urinary tract infection), acute organ failure (kidney, heart, or liver failure), and anemia requiring red blood cell transfusion. The primary outcomes were one-year mortality and residential status following admission. A logistic regression model was used to examine potential influencing factors, including age, Charlson Comorbidity Index, fracture type, pre-fracture living status, discharge destination, and dementia. Mortality and residential status at one year were obtained for all patients through the national citizen registry, ensuring complete follow-up.

Data analysis was performed using SPSS (v25.0, IBM Corp., Armonk, NY, USA). Continuous variables were assessed for normality using visual inspection and the Shapiro-Wilk test. Age showed an approximately normal distribution and is therefore reported as mean ± standard deviation. Categorical variables are presented as absolute and relative frequencies. Kaplan-Meier curves were generated to visually illustrate time-to-event patterns for one-year mortality across fracture types and relevant clinical subgroups.

Before conducting regression analyses, assumptions relevant to logistic regression were considered, including independence of observations, an adequate events-per-variable ratio, absence of relevant multicollinearity among covariates, and an approximately linear relationship between continuous predictors and the outcome. Binary outcomes were analysed using logistic regression, and ordered categorical outcomes using ordinal regression. Femoral neck fractures were chosen as the reference category because they represent the largest and clinically most established fragility fracture group. The dataset was screened for implausible values, and no influential outliers requiring exclusion were identified. The available sample size provided an adequate events-per-variable ratio for the prespecified models. For the primary and secondary categorical outcome measures, logistic and ordinal regression analyses were performed using prespecified covariates to account for potential confounding. A p-value < 0.05 was considered statistically significant.

## Results

### Patient population

The study cohort comprised 486 geriatric patients with fragility fractures (Table 1), with a mean age of 83.8 years (± 6.6). Females accounted for 70.2% (341/486) of the population, and males for 29.8% (145/486).

**Table 1:** Patient population

**Variable**	**Mean/Count**	**SD/per cent**
*n*	486	100%
Age [years]	83.8	± 6.6
Gender		
male	145	30%
female	341	70%
Fracture type		
Femoral neck	117	24%
Trochanteric	146	30%
Periprosthetic femur	26	5%
Cervical spine	25	5%
Thoracolumbar spine	63	13%
Pelvic ring	76	16%
Proximal humerus	33	7%
ISAR score		
2	145	30%
3	151	31%
4	123	25%
5	54	11%
6	13	3%
Charlson Comorbidity Index		
3	29	6%
4	90	19%
5	118	24%
6	99	20%
7	64	13%
8	40	8%
9	28	6%
>10	18	4%
Residential status prior fracture		
Living in own household	355	73%
Nursing facility	131	27%
Dementia	174	36%
Complications		
Nonsurgical	199	41%
Surgical	14	3%

The most common fracture types involved the proximal femur, including trochanteric fractures (30.0%, *n* = 146) and femoral neck fractures (24.1%, *n* = 117). Spinal fractures accounted for 18.1% of all cases (*n* = 88), comprising thoracolumbar spine fractures (13.0%, *n* = 63) and cervical spine fractures (5.1%, *n* = 25). Additional fracture types included pelvic ring fractures (15.6%, *n* = 76), proximal humerus fractures (6.8%, *n* = 33), and periprosthetic proximal femoral fractures (5.3%, *n* = 26).

Prior to the fracture, 73.0% of patients (*n* = 355) were living in their own household, while 27.0% (*n* = 131) resided in a nursing facility. Dementia was present in 35.8% of the cohort (*n* = 174). Complications during the hospital stay were predominantly nonsurgical (40.9%, *n* = 287), with surgical complications being relatively rare (2.9%, *n* = 14).

### Fracture-specific one-year mortality

**Fig. 2:**
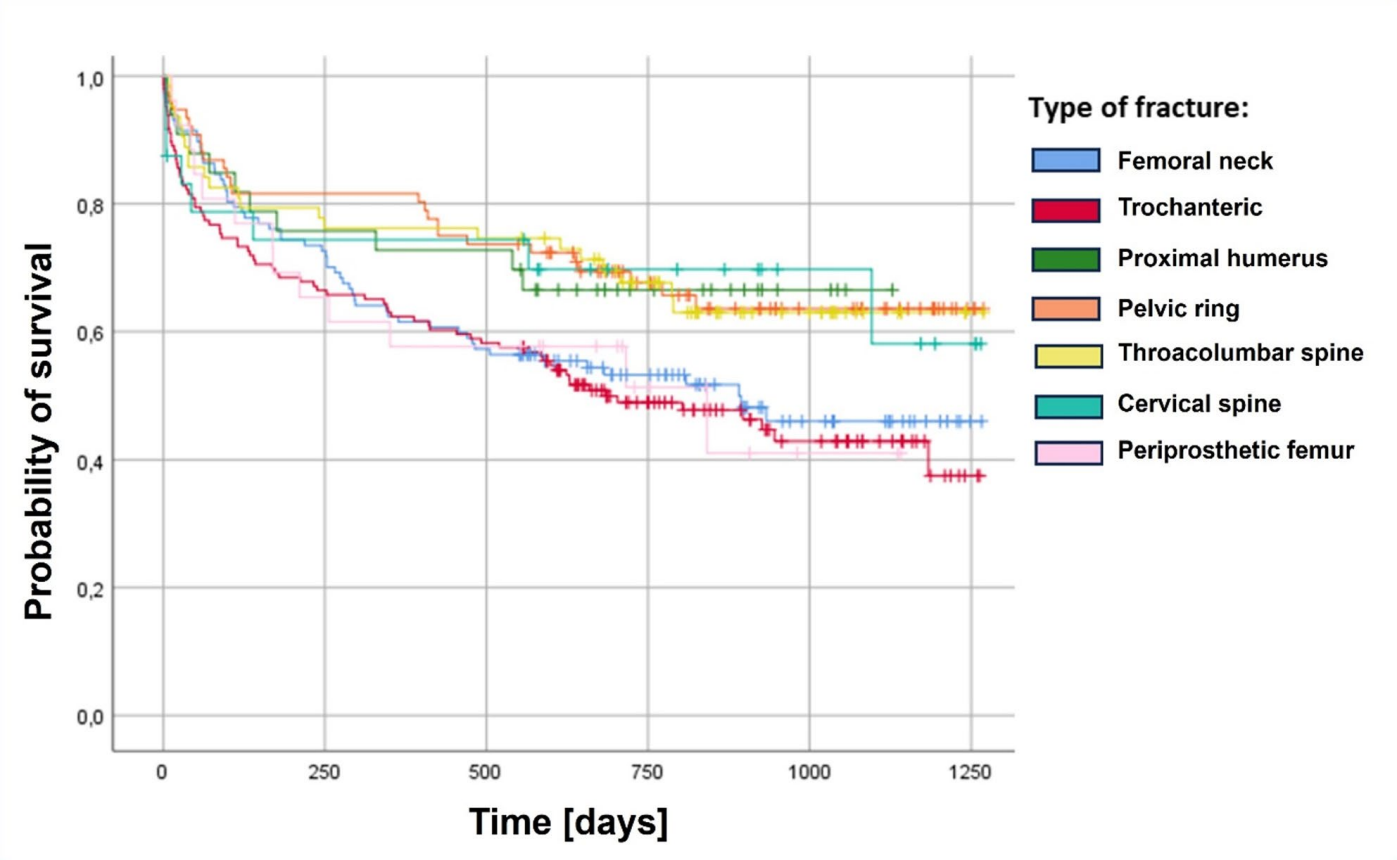
Kaplan–Meier survival curves for different types of fragility fractures

The overall one-year mortality rate in the study cohort was 32.3% (157/486 patients; Table 2). Mortality varied by fracture type (Fig. 2). Among patients with femoral neck fractures 38.5% (45/117) died within one year, compared to 37.7% (55/146) of those with trochanteric fractures. Patients with cervical spine fractures had a mortality rate of 32.0% (8/32), followed by thoracolumbar spine fractures with 23.8% (15/63), and proximal humerus fractures with 27.3% (9/33). The highest mortality was observed in patients with periprosthetic proximal femoral fractures at 42.3% (11/26). In contrast, pelvic ring fractures had the lowest one-year mortality at 18.4% (14/76). In logistic regression analysis, pelvic ring fractures were significantly associated with improved one-year survival (OR 3.46, 95% CI [1.0–11.99], *p* = 0.006), while other fracture types showed no significant association (Table 2).

**Table 2:** One-year mortality for the different types of fragility fractures

Fracture type	Deaths/Patients	One-year mortality [%]
Femoral neck	45/117	38.5
Trochanteric	55/146	37.7
Periprosthetic femur	11/26	42.3
Cervical spine	8/25	32.0
Thoracolumbar spine	15/63	23.8
Pelvic ring	14/76	18.4
Proximal humerus	9/33	27.3
**Total**	**157/486**	**32.3**

### Residential status before and after fracture – functional outcome

**Fig. 3:**
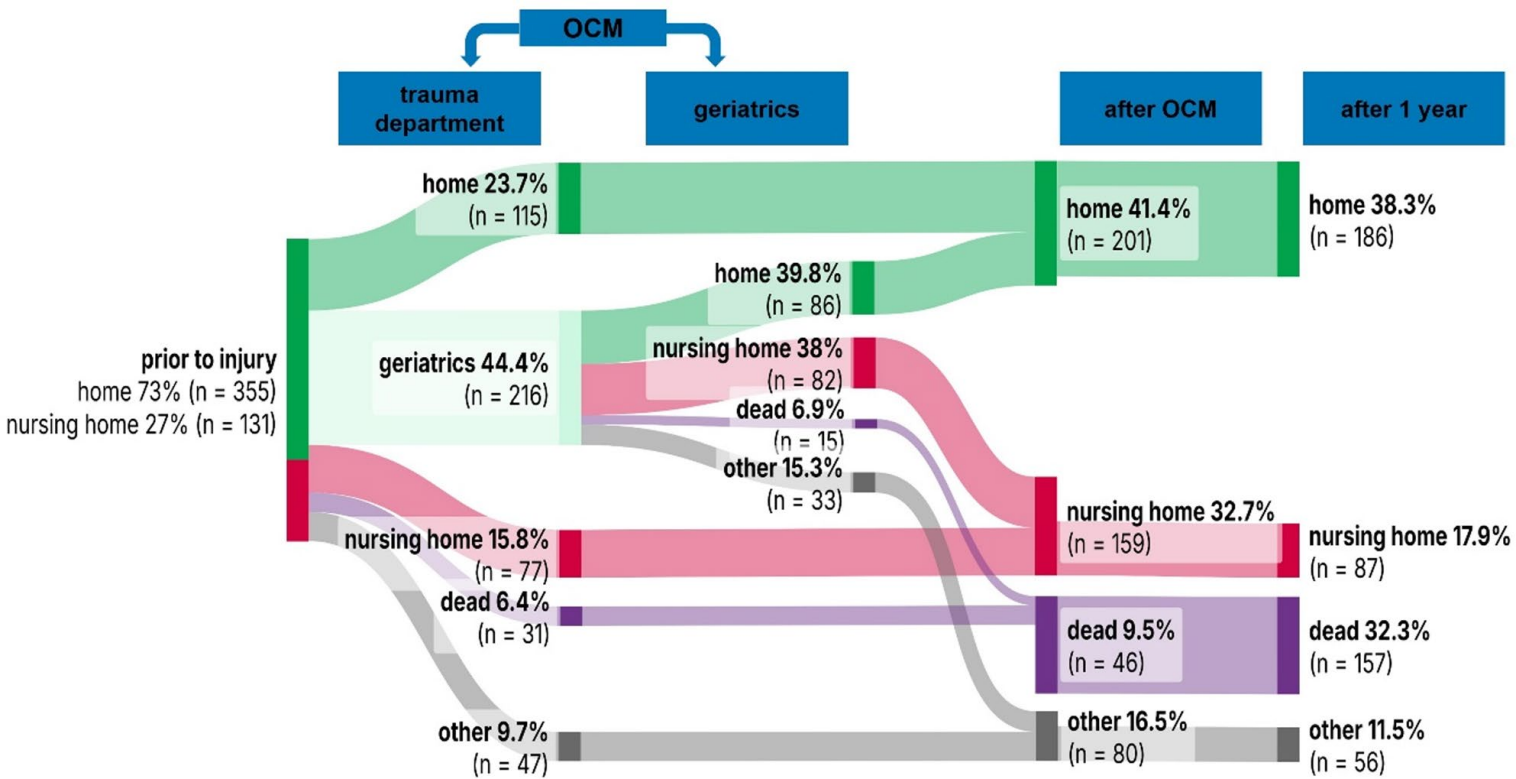
Sankey diagram depicting patient movement over the first year. *OCM: Orthogeriatric Co-Management*

Prior to the fracture, 73.0% of patients (355/486) were living in their own household, while 27.0% (131/486) resided in a nursing facility. One year after the fracture, only 52.3% (186/355) of those previously living at home were still able to maintain independent living. Patient movement and changes in residential status over the course of one year are outlined in Fig. 3.

One-year mortality was lower among those patients previously living at home (26.5%, 94/355) compared to nursing home residents (48.1%, 63/131). This crude difference was statistically significant in unadjusted analysis (χ² = 19.46, *p* < 0.001). However, in logistic regression analysis, pre-fracture residence in a nursing facility was not a significant independent predictor of mortality (OR 0.79, 95% CI [0.63–1.0.63.0] *p* = 0.45; Table 3).

### Impact of discharge destination on mortality

The global logistic regression model was statistically significant (*p* < 0.001) and yielded a Nagelkerke’s R² of 0.209, indicating that the model explained part of the variance in one-year mortality. Discharge destination was strongly associated with survival. Among patients discharged home one-year mortality was 12.9% (26/201). In contrast, patients discharged to a nursing facility had a mortality rate of 39.6% (63/159). Overall, one-year mortality of patients not discharged home was 35.7% (85/238). In logistic regression analysis, discharge to a nursing home remained a significant negative predictor for one-year survival (OR 0.37, 95% CI [0.13–1.0], *p* = 0.003, Table 3). Also, discharge to other institutions (e.g. department of another medical specialty, rehabilitation clinics) was associated with reduced survival (OR 0.43, 95% CI [0.18–1.0], *p* = 0.015; Table 3).

**Fig. 4:**
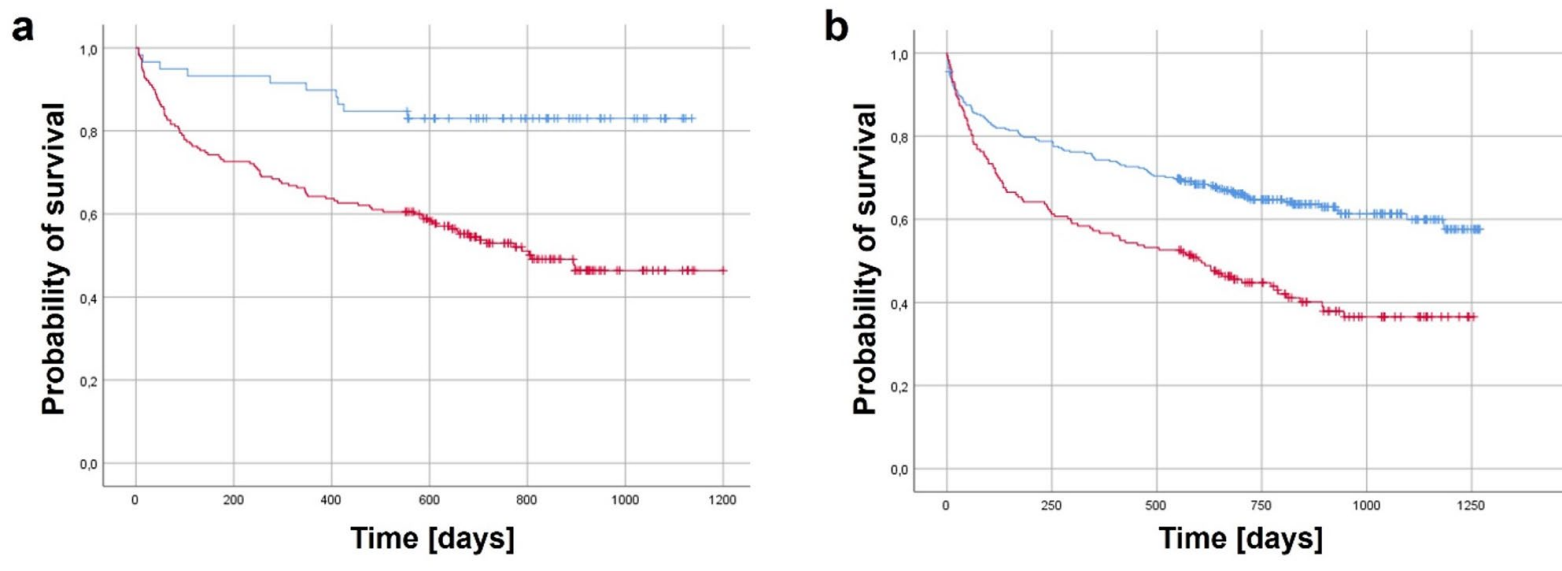
Kaplan–Meier survival curves for Charlson Comorbidity Index and dementia. **a **Survival curves for patients with a Charlson Comorbidity Index (CCI) > 6 (red) versus≤ 6 (blue). **b **Survival curves for patients with (red) versus without (blue) dementia. Censored cases are indicated by tick marks. Logistic regression analysis identified a CCI > 6 as a significant independent predictor of increased one-year mortality (OR 0.44, 95% CI [0.20–1.0], *p* = 0.001)

In addition to discharge destination, patient-related factors also influenced survival. A Charlson Comorbidity Index > 6 was a significant independent predictor of increased mortality (OR 0.44, 95% CI [0.19–1.0], *p* = 0.001). Dementia showed a numerically higher mortality risk (OR 0.64, 95% CI [0.41–1.0], *p* = 0.082; Fig. 4), but this association did not reach statistical significance.

**Table 3:** Logistic regression for one-year survival

Variable	B	OR	95% CI	*p*-value
Fracture type				
Femoral neck^R^				0.127
Trochanteric	0.524	1.688	[1.0, 2.852]	0.090
Periprosthetic femur	0.079	1.082	[1.0, 1.171]	0.874
Cervical spine	0.261	1.298	[1.0, 1.685]	0.674
Thoracolumbar spine	0.827	2.286	[1.0, 5.228]	0.068
** Pelvic ring**	**1.242**	**3.462**	**[1.0, 11.989]**	**0.006**
Proximal humerus	0.403	1.496	[1.0, 2.239]	0.439
Age	− 0.025	0.975	[0.951, 1.0]	0.195
** Charlson Comorbidity Index > 6**	**− 0.811**	**0.444**	**[0.198, 1.0]**	**0.001**
Dementia	− 0.447	0.640	[0.409, 1.0]	0.082
Nursing home resident	− 0.233	0.793	[0.628, 1.0]	0.453
Discharged home^R^				0.019
** Discharge to a nursing home**	**− 1.006**	**0.366**	**[0.134, 1.0]**	**0.003**
** Other discharge modalities**	**− 0.850**	**0.427**	**[0.183, 1.0]**	**0.015**

^R^ Reference category

Boldfaced values indicate significance at *p* < 0.05. B: Regression coefficient; OR: Odds ratio; 95% CI: 95% Confidence interval

## Discussion

This study provides comprehensive one-year outcome data following orthogeriatric co-management of a real-world cohort of patients with various fragility fractures. A key strength lies in the inclusion of multiple fracture types beyond the hip – such as pelvic ring, vertebral, proximal humerus, and periprosthetic femoral fractures – treated within a certified Geriatric Trauma Centre. Furthermore, complete follow-up via the national citizen registry enabled robust assessment of both mortality and functional recovery, operationalized as residential status.

### Fracture-specific mortality: site matters

The overall one-year mortality rate in our cohort was 32.3%, which aligns with prior studies of geriatric trauma patients receiving interdisciplinary care [16]. However, mortality varied substantially across fracture types. Femoral neck fractures showed a one-year mortality of 38.5%, while trochanteric fractures reached 37.7%, confirming the high lethality associated with proximal femoral fractures. Periprosthetic femoral fractures exhibited with 42.3% the highest one-year mortality, likely reflecting increased surgical complexity, prolonged immobility, and higher baseline frailty. In contrast, pelvic ring fractures were associated with the lowest one-year mortality (18.4%) and emerged as the only fracture type independently predictive of improved survival in logistic regression analysis (OR 3.46, *p* = 0.006). These findings may be attributable to the reduced operative invasiveness of percutaneous techniques commonly used in geriatric pelvic surgery, coupled with the high feasibility of early postoperative mobilization.

Similarly, a single-center prospective cohort study of 265 long-term care residents managed under orthogeriatric co-management reported a one-year mortality rate of 29.4%, comparable to our findings. Crucially, this study found no significant difference in mortality between hip and non-hip fragility fractures, underscoring that excess mortality is not confined to hip fractures alone and extends across a broad spectrum of fracture types [16]. Similarly, a large matched-cohort analysis from Canada demonstrated that fragility fractures at any site increase mortality risk among older adults, underlining the systemic impact of these injuries beyond the hip [9]. Together with our data, these findings highlight the urgent need for comprehensive orthogeriatric strategies that address all major fragility fractures, rather than focusing exclusively on the hip.

Considering that the presented study cohort included various fracture types beyond hip fractures, the comparatively high one-year mortality observed in our cohort may partly reflect some selection bias, as only patients aged over 70 years with an ISAR score of 2 or higher were included. The high mortality within this group indicates that routine ISAR screening at admission effectively identifies a particularly vulnerable subgroup likely to benefit from structured orthogeriatric co-management. However, while this observation is noteworthy, our data do not permit comparison with patients with lower ISAR scores and therefore do not allow validation of the tool.

### Discharge destination as a surrogate marker of recovery potential

Discharge destination emerged as one of the strongest predictors of one-year survival. Patients discharged home had a one-year mortality of only 12.9%, compared to 39.6% among those transferred to nursing facilities. Even after adjustment, discharge to a nursing home (OR 2.73, *p* = 0.003) or to other institutional settings (OR 2.34, *p* = 0.015) remained associated with significantly poorer outcomes. This finding reinforces evidence that discharge status serves as a powerful surrogate for functional reserve and available support systems – factors not fully captured by comorbidity indices alone.

Comparable findings were reported from Erivan et al. in a large cohort study of more than 1,200 femoral neck fracture patients, where discharge to home was strongly protective (HR 0.30), while discharge to a nursing home markedly increased mortality risk (HR 1.82) over three years [17]. Similarly, Deemer et al. analysed over 2,500 hip fracture patients and found that those discharged home were healthier, less dependent on assistive devices, and had lower readmission and complication rates [18].

Together with our data, these studies highlight discharge destination as a clinically meaningful proxy for recovery potential and long-term prognosis, as it reflects underlying factors such as functional reserve, frailty, and available support systems that are not fully captured by comorbidity measures alone. Nonetheless, these associations should not be interpreted as causal relationships. Discharge destination likely reflects these underlying characteristics rather than exerting an independent effect on mortality. As our model did not include a formal frailty measure, residual confounding cannot be excluded. Moreover, potential collinearity between predictors such as comorbidity, cognitive impairment, and discharge destination may have influenced the effect estimates. Therefore, the findings should be interpreted as associative signals within a real-world geriatric cohort rather than causal determinants.

### Comorbidity and dementia: consistent but nuanced predictors

Patient-related factors also shaped outcomes. A Charlson Comorbidity Index > 6 was an independent predictor of increased one-year mortality (OR 2.52, *p* = 0.001), confirming the established link between comorbidity burden and poor prognosis in geriatric trauma care [19]. While dementia was associated with a numerically higher mortality risk (OR 1.56, *p* = 0.082), it did not reach statistical significance, possibly due to the confounding influence of discharge disposition and the high baseline mortality among cognitively impaired patients. This finding aligns with a large Swedish registry study by Forssten et al., which analysed 121,305 patients with hip fractures and demonstrated that dementia loses predictive power for postoperative mortality once frailty measures such as prefracture mobility and institutionalisation are incorporated into the model [20]. Their analysis, together with our data, may support the concept that dementia primarily acts as a surrogate for frailty rather than an independent predictor of mortality.

### Functional recovery: identifying orthogeriatric responders

Beyond survival, maintenance of functional independence represents a critical outcome for older adults. In our cohort, only 52.3% of patients who were living independently prior to the fracture returned to their own household one year later. While this decline highlights the long-term burden of fragility fractures of all types, it also underscores the therapeutic potential of targeted orthogeriatric management.

To better capture this dimension, we propose the concept of the *orthogeriatric responders*: patients who, through structured interdisciplinary management, are able to return to their pre-fracture functional level, while acknowledging that many older adults may appropriately follow supportive or palliative functional trajectories due to progressive frailty. Future research should aim to identify modifiable predictors of this outcome – such as early mobilization, cognitive stability, and the feasibility of discharge home – and to incorporate patient-centered outcomes, including quality of life.

### Clinical implications

The findings of this study offer several practical implications for orthogeriatric trauma care across a broad spectrum of fragility fractures, not limited to hip fractures. First, routine ISAR screening at admission appears useful for early identification of vulnerable older adults who may benefit from structured interdisciplinary management. Second, the strong association between discharge destination and one-year outcomes highlights the importance of early, coordinated discharge planning, including efforts to enable discharge home when feasible and safe. Third, factors associated with more favourable functional trajectories – such as early mobilization and cognitive stability – may serve as targets for optimizing in-hospital care pathways. Finally, the substantial decline in functional independence observed across all fracture types underscores the need to integrate patient-centered outcomes, including quality of life, into care planning and follow-up. Together, these implications may help refine orthogeriatric pathways and guide resource allocation for the full range of fragility fractures encountered in clinical practice.

## Limitations

This study has several limitations. Its retrospective, single-centre design limits causal inference and may reduce generalizability, particularly to healthcare systems without certified orthogeriatric structures. Detailed data on rehabilitation practices and functional outcomes beyond residential status were not available. Moreover, the pre-fracture residential status variable lacked granularity with regard to formal and informal support, which may have introduced unmeasured variability. These aspects should be considered when interpreting the findings.

## Conclusions

Fragility fractures, irrespective of anatomical location, are associated with high one-year mortality and substantial functional decline. These findings highlight the need for orthogeriatric strategies that extend beyond hip fracture care and address the full spectrum of fragility fractures. Discharge home emerged as a strong surrogate marker of survival. Future studies should identify modifiable factors within structured interdisciplinary care that support recovery to pre-fracture functional levels and help further define *orthogeriatric responders*.

## Data Availability

The datasets used and/or analysed during the current study are available from the corresponding author on reasonable request.

## Abbreviations

CCI: Charlson Comorbidity Index
CI: Confidence Interval
DGU: Deutsche Gesellschaft für Unfallchirurgie (German Society for Trauma Surgery)
ISAR: Identification of Seniors at Risk
OR: Odds Ratio
